# Nonsynonymous variants in *MYH9* and *ABCA4* are the most frequent risk loci associated with nonsyndromic orofacial cleft in Taiwanese population

**DOI:** 10.1186/s12881-016-0322-2

**Published:** 2016-08-15

**Authors:** Hsiu-Huei Peng, Nai-Chung Chang, Kuo-Ting Chen, Jang-Jih Lu, Pi-Yueh Chang, Shih-Cheng Chang, Yah-Huei Wu-Chou, Yi-Ting Chou, Wanni Phang, Po-Jen Cheng

**Affiliations:** 1 Department of Obstetrics and Gynecology, Chang Gung Memorial Hospital, Linkou Medical Center, Taoyuan, Taiwan; 2 Chang Gung University College of Medicine, Taoyuan, Taiwan; 3 Department of Laboratory Medicine, Chang Gung Memorial Hospital, Linkou Medical Center, Taoyuan, Taiwan; 4 Department of Plastic and Reconstructive surgery, Chang Gung Memorial Hospital, Linkou Medical Center, Taoyuan, Taiwan; 5 Human Molecular Genetics Laboratory, Department of Medical Research, Chang Gung Memorial Hospital, Linkou Medical Center, Taoyuan, Taiwan

**Keywords:** Nonsyndromic orofacial clefts, Next-generation sequencing, MYH9, *ABCA4*

## Abstract

**Background:**

Nonsyndromic orofacial cleft is a common birth defect with a complex etiology, including multiple genetic and environmental risk factors. Recent whole genome analyses suggested associations between nonsyndromic orofacial cleft and up to 18 genetic risk loci (*ABCA4*, *BMP4*, *CRISPLD2*, *GSTT1*, *FGF8*, *FGFR2*, *FOXE1*, *IRF6*, *MAFB*, *MSX1*, *MTHFR*, *MYH9*, *PDGFC*, *PVRL1*, *SUMO1*, *TGFA*, *TGFB3*, and *VAX1*), each of which confers a different relative risk in different populations. We evaluate the nonsynonymous variants in these 18 genetic risk loci in nonsyndromic orofacial clefts and normal controls to clarify the specific variants in Taiwanese population.

**Methods:**

We evaluated these 18 genetic risk loci in 103 cases of nonsyndromic orofacial clefts and 100 normal controls using a next-generation sequencing (NGS) customized panel and manipulated a whole-exon targeted-sequencing study based on the NGS system of an Ion Torrent Personal Genome Machine (IT-PGM). IT-PGM data processing, including alignment with the human genome build 19 reference genome (hg19), base calling, trimming of barcoded adapter sequences, and filtering of poor signal reads, was performed using the IT platform-specific pipeline software Torrent Suite, version 4.2, with the plug-in “variant caller” program. Further advanced annotation was facilitated by uploading the exported VCF file from Variant Caller to the commercial software package Ion Reporter; the free online annotation software Vanno and Mutation Taster. Benign or tolerated amino acid changes were excluded after analysis using sorting intolerant from tolerant and polymorphism phenotyping. Sanger sequencing was used to validate the significant variants identified by NGS. Furthermore, each variant was confirmed in asymptomatic controls using the Sequenom MassARRAY (San Diego, CA, USA).

**Results:**

We identified totally 22 types of nonsynonymous variants specific in nonsyndromic orofacial clefts, including 19 single nucleotide variants, 2 deletions, and 1 duplication in 10 studied genes(*ABCA4*, *MYH9*, *MTHFR*, *CRISPLD2*, *FGF8*, *PVRL1*, *FOXE1*, *VAX1*, *FGFR2*, and *IRF6)*. Nonsynonymous variants in *MYH9* and *ABCA4*, which were detected in 6 and 5 individuals, respectively, were identified to be the most frequent risk loci in nonsyndromic orofacial clefts in the Taiwanese population.

**Conclusions:**

Nonsynonymous variants in *MYH9* and *ABCA4* were identified to be the most frequent risk loci in nonsyndromic orofacial clefts in the Taiwanese population. These findings in our study have provided additional information regarding specific variants associated with nonsyndromic orofacial clefts in different population and demonstrate the power of our customized NGS panel, which is clinically useful for the simultaneous detection of multiple genes associated with nonsyndromic orofacial clefts.

**Electronic supplementary material:**

The online version of this article (doi:10.1186/s12881-016-0322-2) contains supplementary material, which is available to authorized users.

## Background

Nonsyndromic orofacial clefts, which include cleft lip with or without cleft palate (CL/P) and isolated cleft palate (CPI), are among the most common congenital malformations worldwide. The prevalence of this major birth defect widely ranges from 1/700 to 1/1000, with fluctuations attributed to different areas and ethnicities [1, 2]. Epidemiological data reveal that the prevalence of nonsyndromic CL/P is lowest among African populations (0.4/1000), intermediate among European populations (1/1000), and highest among Asian populations (2/1000) [3]. Nonsyndromic orofacial cleft is not only associated with increased infant morbidity and mortality but also has enormous effects on speech, hearing, appearance, and mental disability, thereby increasing long-term medical costs and placing substantive burdens on families and societies [4, 5].

Nonsyndromic orofacial cleft is an etiologically heterogeneous disease with multiple genetic and environmental risk factors [6]. Maternal smoking, alcohol consumption, and folate and vitamin deficiencies, particularly during the first trimester of pregnancy, have been suggested to increase the occurrence of nonsyndromic CL/P [7, 8]. Previous gene identification studies of nonsyndromic CL/P were generally based on genome-wide association studies (GWASs) [9–12], genome-wide linkage studies [13], and GWAS meta-analyses [14, 15]. These studies identified genetic risk loci associated with nonsyndromic CL/P on chromosomes 1p22, 1p36, 2p21, 3p11.1, 8q21.3, 8q24, 9q22, 10q25, 15q22, 17p13, 17q22, and 20q12.

Although GWAS studies have identified multiple nonsyndromic CL/P-associated genetic loci, further progress in the identification of casual variants has been limited because these approaches focus on common variants and neglect low-frequency variants [16]. To identify novel and low-frequency variants, next-generation sequencing (NGS)-based techniques facilitate the simultaneous detection of causal variants in large genomic regions. We selected 18 nonsyndromic orofacial cleft-related candidate genes, including *ABCA4*, *BMP4*, *CRISPLD2*, *GSTT1*, *FGF8*, *FGFR2*, *FOXE1*, *IRF6*, *MAFB*, *MSX1*, *MTHFR*, *MYH9*, *PDGFC*, *PVRL1*, *SUMO1*, *TGFA*, *TGFB3,* and *VAX1*, based on previous associated studies [7, 17] to conduct a customized NGS panel and subsequently manipulated a whole-exon targeted-sequencing study based on the NGS system of an Ion Torrent Personal Genome Machine (IT-PGM). A total of 103 patients with nonsyndromic orofacial clefts and 100 independent asymptomatic normal controls were enrolled to investigate potential variants associated with nonsyndromic orofacial clefts and identify specific nonsynonymous variants in Taiwanese population.

## Methods

### Case enrollment and ethics statement

The study population included 103 Taiwanese patients with isolated, nonsyndromic orofacial clefts recruited from the Linkou Chang Gung Memorial Hospital from 1995 to 2014; patients’ diagnoses had been confirmed via neonatal photographs or chart descriptions written by plastic surgeons or clinical geneticists. Individuals with other systemic abnormalities, developmental or mental delays, and confirmed chromosomal abnormalities were excluded from this study. For the control group, we recruited 100 asymptomatic Taiwanese volunteers who had no family history of orofacial clefts from among the employees of Linkou Chang Gung Memorial Hospital.

This study was approved by the institutional review board (IRB 101-4637A3) at Linkou Chang Gung memorial hospital, and written informed consent was obtained from all adult participants and the parents or guardians of pediatric participants.

### Sample collection and genomic DNA preparation

Genomic DNA was extracted from 10-ml peripheral blood samples using the QIAamp DNA Blood Mini Kit (Qiagen, Valencia, CA, USA) as follows: 20 μL of QIAGEN Protease (or protease K) was mixed with 200 μL of buffy coat via a 15-s vortex step. The mixture was then incubated at 56 °C for 10 min after adding 200 μL of Buffer AL. Next, 200 μL of 96–100 % ethanol was added, followed by a 15-s vortex and transfer to a QIAamp Mini spin column. The silica membrane was washed via centrifugation with Buffers AW1 and AW2. Genomic DNA was eluted with Buffer AE, and the quantity and quality were determined using a Nanodrop (Thermo Fisher Scientific, Waltham, MA, USA) and Qubit 2.0 fluorometer (Life Technologies, Carlsbad, CA, USA).

### Design of a customized NGS panel for nonsyndromic orofacial cleft

We analyzed 18 genetic loci associated with the risk of nonsyndromic orofacial cleft, including *IRF6*, *VAX1*, *ABCA4*, *BMP4*, *FGFR2*, *FOXE1*, *MAFB*, *MSX1*, *MYH9*, *CRISPLD2*, *FGF8*, *GSTT1*, *MTHFR*, *PDGFC*, *PVRL1*, *SUMO1*, *TGFA*, and *TGFB3* (Table 1). We used the Ion AmpliSeq™ Designer v2.2.1 cloud-based software program, which was supplied free of charge by Life Technologies, to design our customized panel. Moreover, we used the National Center for Biotechnology Information (NCBI) ClinVar database to identify pathogenic variants in these 18 genes and set up a hotspot database.

**Table 1:** List of 18 selected genes studied in patients with nonsyndromic orofacial clefts and normal controls

Gene	Gene size (bp)	Map location	Protein
*ABCA4*	128,313	1p21-p22.1	ATP-binding cassette, sub-family A, member 4
*BMP4*	9026	6p12	Bone morphogenetic protein 4
*CRISPLD2*	100,788	16q24.1	Cysteine-rich secretory protein LCCL domain containing 2
*GSTT1*	8548	22q11.23	Glutathione S-transferase theta 1
*FGF8*	10,240	10q24.32	Fibroblast growth factor 8
*FGFR2*	120,129	10q26.13	Fibroblast growth factor receptor 2
*FOXE1*	3462	9q22	Forkhead box E1
*IRF6*	20,553	1q32.2	Interferon regulatory factor 6
*MAFB*	3393	20q12	V-maf avian musculoaponeurotic fibrosarcoma oncogene homolog B
*MSX1*	4272	4p16.2	Msh homeobox 1
*MTHFR*	21,198	1p36.22	Methylenetetrahydrofolate reductase
*MYH9*	106,741	22q12.3	Myosin, heavy chain 9
*PDGFC*	210,941	4q32	Platelet derived growth factor C
*PVRL1*	105,675	11q23.	Poliovirus receptor-related 1
*SUMO1*	32,429	17p13.1	Small ubiquitin-like modifier 1
*TGFA*	106,914	6p21.3	Transforming growth factor, alpha
*TGFB3*	24,893	14q24.3	Transforming growth factor, beta 3
*VAX1*	9781	10q25.3	Ventral anterior homeobox 1

### IT-PGM AmpliSeq library preparation and IT-PGM sequencing

AmpliSeq multiplexed libraries were constructed using the Ion AmpliSeq Library Kit 2.0 according to the manufacturer’s protocol (Life Technologies, Part #4475345), with some modifications. The preparation was started with 30 ng of genomic DNA in a volume of ≤6 μL; the 501 amplicons were amplified by PCR and divided into two primer pools. We increased the annealing and extension steps of PCR program from 4 to 8 min to improve the efficiency of longer amplicons. Primer sequences were partially digested with FuPa reagent, and barcoded adapters were ligated with DNA ligase. Following purification and size selection using AMPure beads (Beckman Coulter, Brea, CA, USA), the prepared library was quantified using a Qubit 2.0 Fluorometer (Life Technologies) and Bioanalyzer high-sensitivity DNA chip (Agilent Technologies Inc., Santa Clara, CA, USA). Quantified libraries were pooled and diluted further to generate a 10-pmol/L working stock. To clonally amplify library DNA onto IonSpheres (ISPs), we used emulsion PCR, emulsion breaking, and template enrichment using the Ion OneTouch™ 200 system and Template Kit v2.0 (Life Technologies) according to the manufacturer’s protocols. Enriched ISPs were prepared for sequencing using the Ion PGM 200 Sequencing Kit v2.0 and loaded on an Ion 316 chip v2 or Ion 318 chip v2, depending on whether 7 or 14 samples were to be sequenced, respectively. To sequence an authentic variant, an ideal average coverage for each amplicon of 500× and variant frequency of at least 5 % in the wild-type background were used in this study.

### Bioinformatics analysis

IT-PGM data processing, including alignment with the human genome build 19 reference genome (hg19), base calling, trimming of barcoded adapter sequences, and filtering of poor signal reads, was performed using the IT platform-specific pipeline software Torrent Suite, version 4.2, with the plug-in “variant caller” program (Life Technologies). Further advanced annotation was facilitated by uploading the exported VCF file from Variant Caller to the commercial software package Ion Reporter (Life Technologies); the free online annotation software Vanno [18] and MutationTaster. Benign or tolerated amino acid changes were excluded after analysis using sorting intolerant from tolerant (SIFT) and polymorphism phenotyping (PolyPhen). In addition, we used the Integrative Genomics Viewer to visualize the status of each read alignment and the presence of variants from the reference genome to clarify possible strand biases or sequencing errors.

### Experimental validation

Validation by alternative sequencing methods was required for NGS-identified variants that passed the in-house filtering steps. Sanger sequencing was used to validate the significant variants identified by NGS. Furthermore, each variant was confirmed in asymptomatic controls using the Sequenom MassARRAY (San Diego, CA, USA).

## Results

### Cases

The clinical features of the 103 patients with nonsyndromic orofacial clefts and 100 normal controls are listed in Table 2.

**Table 2:** Clinical characteristics of patients with nonsyndromic orofacial clefts and normal controls

Characteristics		Nonsyndromic orofacial clefts	Normal controls
		Number (%)	Number (%)
Gender	Male	48 (46.6 %)	45 (45.0 %)
	Female	55 (53.4 %)	55 (55.0 %)
Age	Range	1–41	21–58
Cleft type	Cleft lip only	12 (11.6 %)	absent
	Cleft palate only	32 (31.0 %)	absent
	Cleft lip and palate	56 (54.4 %)	absent
	Unclassified	3 (3.0 %)	absent
Cleft site	Unilateral	53 (51.4 %)	absent
	Bilateral	46 (44.7 %)	absent
	Unclassified	4 (3.9 %)	absent

### Customized NGS panel for nonsyndromic orofacial cleft

Detailed information about this customized panel is listed in Additional file 1: Table S1. This panel comprises 501 amplicons divided into two primer pools: 254 amplicons in primer pool 1 and 247 amplicons in primer pool 2. The amplicon sizes are 125–275 bp. Details regarding the numbers of exons and amplicons in the 18 selected genes are listed in Additional file 1: Table S2. The average target region coverage rate was 94.09 %.

### Performance of the customized NGS panel

**Fig. 1:**
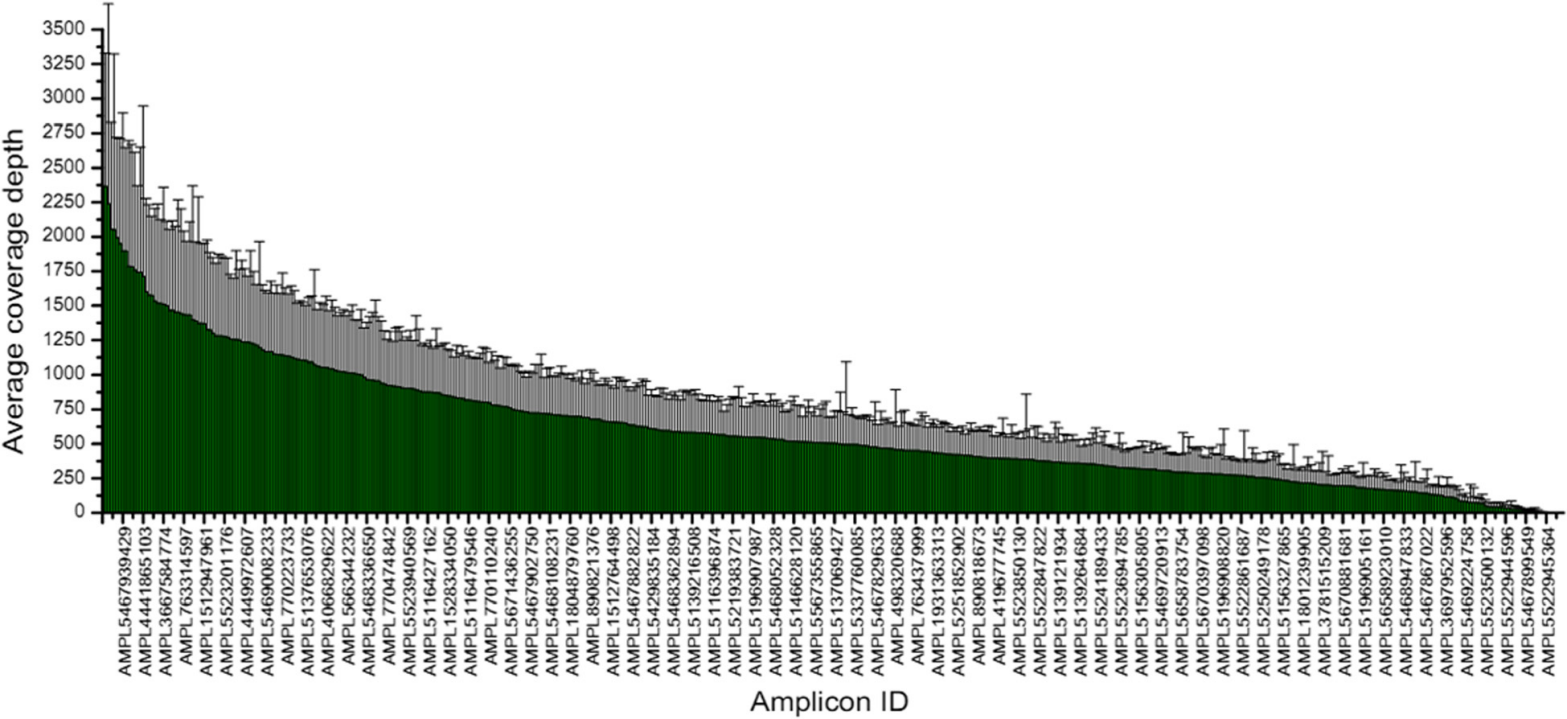
Nonsyndromic orofacial clefts customized next-generation sequencing panel performance with an average coverage depth of 501 amplicons

Our quality control standard for defining a true variant is a gene locus coverage depth >50×. In other words, the average coverage depth of each amplicon should receive more than 50× reads to reduce the risk of misjudgment in subsequent PGM sequencing. Figure 1 shows the average coverage depths of the 501 amplicons in the 203 evaluated samples (103 nonsyndromic orofacial cleft and 100 normal control samples); 95 % of amplicons had a gene locus coverage depth >50 × .

### All nonsynonymous variants in patients with nonsyndromic orofacial clefts

The distributions of all nonsynonymous variants of the 18 analyzed genes among patients with nonsyndromic orofacial clefts are shown in Additional file 1: Table S3. We identified 29 types of nonsynonymous variants, including 23 single nucleotide variants, 4 deletions, 1 duplication, and 1 insertion.

### All nonsynonymous variants in normal controls

The distributions of all nonsynonymous variants of the 18 analyzed genes among normal controls are shown in Additional file 1: Table S4. We identified 15 types of nonsynonymous variants, including 12 single nucleotide variants, 2 deletions, and 1 insertion.

### Specific nonsynonymous variants in patients with nonsyndromic orofacial cleft

**Fig. 2:**
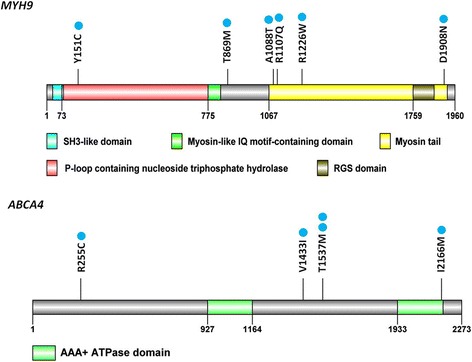
Distribution of nonsynonymous variants specific to orofacial clefts in MYH9 and ABCA4 (blue dots indicate the numbers of affected individuals)

A comparison of nonsynonymous variants between patients with nonsyndromic orofacial clefts and normal controls identified 22 types of specific variants within 10 studied genes in the former group (Table 3). Among these, 19 were single-nucleotide variants (in *ABCA4*, *MYH9*, *MTHFR*, *CRISPLD2*, *FGF8*, *PVRL1*, *FOXE1*, and *FGFR2*), 2 were deletions (in *CRISPLD2* and *IRF6*), and 1 was a duplication (*VAX1*). The nonsynonymous variants in *MYH9* and *ABCA4*, which affected 6 and 5 individuals with nonsynonymous variants, respectively, were the most frequent risk loci among this Taiwanese population. The distributions of nonsynonymous variants in *MYH9* and *ABCA4* are shown in Fig. 2.

**Table 3:** Specific variants found to be associated with nonsyndromic orofacial cleft

Gene	Coding	Amino acid change	PolyPhen	SIFT	Mutation Taster	Phenotype	Number of cases
*MTHFR*	c.1816C > T	p.R606C	Possibly damaging	Damaging	Disease causing	BL CLP	1/103
*MTHFR*	c.62G > A	p.S21N	Benign	Damaging	Disease causing	BL CP	1/103
*MYH9*	c.5722G > A	p.D1908N	Possibly damaging	Damaging	Disease causing	L CLP	1/103
*MYH9*	c.3676C > T	p.R1226W	Benign	Damaging	Disease causing	L CLP	1/103
*MYH9*	c.3320G > A	p.R1107Q	Possibly damaging	Damaging	Disease causing	BL CP	1/103
*MYH9*	c.3262G > A	p.A1088T	Possibly damaging	Damaging	Disease causing	R CL	1/103
*MYH9*	c.2606C > T	p.T869M	Benign	Damaging	Polymorphism	BL CP	1/103
*MYH9*	c.452A > G	p.Y151C	Possibly damaging	Damaging	Disease causing	L CLP	1/103
*CRISPLD2*	c.119_121del	p.40_41del	—	—	Polymorphism	L CLP	1/103
*CRISPLD2*	c.1337C > G	p.A446G	Possibly damaging	Damaging	Disease causing	L CLP	1/103
*ABCA4*	c.6498C > G	p.I2166M	Possibly damaging	Tolerated	Disease causing	R CLP	1/103
*ABCA4*	c.4610C > T	p.T1537M	Possibly damaging	Damaging	Disease causing	L CL; unknown CP	2/103
*ABCA4*	c.4297G > A	p.V1433I	Possibly damaging	Tolerated	Polymorphism	BL CLP	1/103
*ABCA4*	c.763C > T	p.R255C	Possibly damaging	Tolerated	Disease causing	L CL	1/103
*FOXE1*	c.1090G > A	p.G364S	Possibly damaging	Tolerated	Disease causing	BL CLP	2/103
*FGF8*	c.251C > T	p.P84L	Possibly damaging	Damaging	Disease causing	L CL	1/103
*FGF8*	c.250C > T	p.P84S	Possibly damaging	Damaging	Disease causing	L CL	1/103
*VAX1*	c.363dupT	p.C122fs	—	—	Disease causing	R CLP	1/103
*PVRL1*	c.334 T > A	p.S112T	Benign	—	Disease causing	Unknown CLP	1/103
*PVRL1*	c.52C > T	p.L18F	Benign	—	Polymorphism	R CLP; L CLP	2/103
*FGFR2*	c.293C > T	p.T98M	Possibly damaging	Damaging	Disease causing	L CL	1/103
*IRF6*	c.421_423del	p.141_141del	—	—	Disease causing	R CLP	1/103

*BL* bilateral, *CL* cleft lip, *CLP* cleft lip with palate, *CP* cleft palate, *L* left, *R* right

It is worth noticing that in our population, no nonsynonymous variants were found in eight genes (*BMP4*, *GSTT1*, *MAFB*, *MSX1*, *PDGFC*, *SUMO1*, *TGFA*, and *TGFB3*). These eight genes appear to have a weaker association with nonsyndromic orofacial cleft in the Taiwanese population. As most hotspot variants in the NCBI database were previously collected from foreign populations, our findings may reflect specific variants associated with nonsyndromic orofacial cleft in the Taiwanese population.

## Discussion

Orofacial cleft is among the most common human congenital malformations, affecting 135,000 neonates worldwide each year [2]. Orofacial clefts have been associated with both environmental and genetic risk factors and the prevalence of this major birth defect widely ranges among different areas and ethnicities.

In the last decade, major advances in the identification of the causative genetic mutations underlying syndromic forms of CL/P have revealed more than 60 syndromic cleft-associated genes [19]. By contrast, the genetic cause of nonsyndromic forms of CL/P remains mostly unclear. The lack of progress in our understanding of the genetic etiology of nonsyndromic CL/P is obviously associated with the lack of a recognizable mode of inheritance and reduced penetrance of these patients, as well as the low rate of positive family history among affected persons [20]. With the advances in the genomic era, the recent development of powerful and cost-effective genomic tools has opened new routes for phenotyping nonsyndromic orofacial clefts. Recent GWAS [9–12], genome-wide linkage studies [13], and GWAS meta-analyses [14, 15] have suggested that nonsyndromic orofacial clefts might be associated with up to 18 genetic loci, each with a different relative risk in different populations. These candidate loci include *IRF6* (1q32.3-q41), *VAX1* (10q26.1), *ABCA4* (1p22.1-p21), *BMP4* (14q22-q23), *FGFR2* (10q26), *FOXE1* (9q22), *MAFB* (20q11,2-q13.1), *MSX1* (4p16.3-p16.1), *MYH9* (22q13.1), *CRISPLD2* (16q24.1), *FGF8* (10q24), *GSTT1* (22q11.23), *MTHFR* (1p36.3), *PDGFC* (4q32), *PVRL1* (11q23.3), *SUMO1* (2q33), *TGFA* (2p13), and *TGFB3* (14q24) [19, 21–28]. These genetic risk loci carry a different relative risk in different populations. Our study is the first to analyze these 18 genetic loci associated with nonsyndromic orofacial cleft in the Taiwanese population.

As nonsyndromic orofacial cleft is a complex condition affected by multiple genes, genetic testing must be robust and cover a wide spectrum of potential mutations. Unlike traditional sequencing, which screens one gene at a time, exon by exon, NGS techniques allow massive parallel sequencing of as many genes as desired, thereby leveling the economic and technological barriers to detecting mutations on a genome-wide scale. Although this technology is suitable for the detection of any mutation within a rational target, targeted NGS is considered to be particularly useful for detecting mutations in disorders with a highly heterogeneous genetic background. With this understanding, we used a customized NGS panel to rapidly detect possible variants in these 18 statistically validated candidate genes among patients with nonsyndromic orofacial clefts.

In our study, we evaluated 18 genetic risk loci in 103 cases of nonsyndromic orofacial clefts and 100 normal controls from the Taiwanese population using customized NGS, which revealed 22 types of specific variants within 10 studied genes in individuals with nonsyndromic orofacial clefts. Among these, 19 were single nucleotide variants (in *ABCA4*, *MYH9*, *MTHFR*, *CRISPLD2*, *FGF8*, *PVRL1*, *FOXE1*, and *FGFR2*), 2 were deletions (in *CRISPLD2* and *IRF6* gene), and 1 was a duplication (*VAX1*). The nonsynonymous variants in *MYH9* and *ABCA4*, which were detected in 6 and 5 individuals, respectively, were the most frequent risk loci in our Taiwanese population.

MYH9, or myosin heavy chain 9, has been shown to associate with nonsyndromic CL/P in several populations [29]. Abundant, specific expression of MYH9 was observed in the epithelial cells of palatal shelves prior to fusion. The expression level of MYH9 was shown to decrease and be restricted to epithelial triangles before disappearing upon the completion of fusion [30]. In our study, *MYH9* is the most frequent risk loci in the Taiwanese population, providing further evidence for the involvement of MYH9 in the etiology of nonsyndromic CL/P.

A recent GWAS of several populations revealed markers in/near the gene encoding *ABCA4*, indicating a novel susceptibility locus for CL/P [9]. In Honduran and Colombian populations, *ABCA4* is a candidate gene associated with nonsyndromic orofacial clefting [31]. In the Brazilian population, *ABCA4* rs540426 associated strongly with CL/P, unilateral and right CL/P, and bilateral CL/P, whereas the SNP rs481931 exhibited borderline associations with CL/P and bilateral CL/P [32]. However, in a Chinese Han population, *ABCA4* was not found to associated with nonsyndromic orofacial clefts [33]. In our study, several nonsynonymous variants in *ABCA4* were specifically found in individuals with nonsyndromic orofacial clefts from a Taiwanese population.

## Conclusions

Nonsynonymous variants in MYH9 and ABCA4 were identified to be the most frequent risk loci in nonsyndromic orofacial clefts in the Taiwanese population. Our findings provide us more information about specific variants associated with nonsyndromic orofacial clefts in different population, as well as demonstrate the power of our customized NGS panel, which is clinically useful for the simultaneous detection of multiple genes associated with nonsyndromic orofacial clefts. Furthermore, recent NGS studies have shown that fetal DNA from a few milliliters of maternal plasma is sufficient for fetal whole genome sequencing. Importantly, using parental genomes as guides, fetal genome sequences could be scanned for mutations prenatally and noninvasively [34, 35]. In the near future, it will be possible to predict whether a fetus will be affected by a nonsyndromic orofacial cleft based on a targeted NGS-based investigation of genetic risk loci.

## Additional file

Additional file 1: Table S1. Customized NGS panel information for nonsyndromic orofacial clefts. **Table S2.** Detailed panel information about the 18 selected genes studied in nonsyndromic orofacial clefts. **Table S3.** All nonsynonymous variants found in individuals with nonsyndromic orofacial clefts. **Table S4.** All nonsynonymous variants found in normal controls. (DOC 164 kb)
