# Supplementary material for: Nonsynonymous variants in MYH9 and ABCA4 are the most frequent risk loci associated with nonsyndromic orofacial cleft in Taiwanese population
Source: BMC Med Genet. 2016 Aug 15;17:59. doi: 10.1186/s12881-016-0322-2 (PMC4986225; doi:10.1186/s12881-016-0322-2)
Supplement: Additional file 1: Table S1. — Customized NGS panel information for nonsyndromic orofacial clefts. Table S2. Detailed panel information about the 18 selected genes studied in nonsyndromic orofacial clefts. Table S3. All nonsynonymous variants found in individuals with nonsyndromic orofacial clefts. Table S4. All nonsynonymous variants found in normal controls. (DOC 164 kb) [file 12881_2016_322_MOESM1_ESM.doc]

**Additional file 1**

**Table S1 Customized NGS panel information for nonsyndromic orofacial clefts**

| Panel | Nonsyndromic orofacial clefts customized NGS panel |
| --- | --- |
| Design tool | Ion AmpliSeq™ Designer. v2.2.1 |
| Panel size | 80.95 kb |
| Primer pools | 2 |
| Coverage | 94.09% |
| Amplicon size | 125–275 bp |
| Amplicon number | 501 (pool 1: 254; pool 2: 247) |

NGS, next-generation sequencing.

**Table S2 Detailed panel information about the 18 selected genes studied in nonsyndromic orofacial clefts**

| Gene | Number of exons | Number of amplicons | Total bases (bp) | Missed bases (bp) | Covered (%) |
| --- | --- | --- | --- | --- | --- |
| *ABCA4* | 50 | 67 | 8,375 | 21 | 99.75 |
| *BMP4* | 6 | 16 | 2,277 | 0 | 100 |
| *CRISPLD2* | 15 | 31 | 4,904 | 383 | 92.19 |
| *GSTT1* | 5 | 9 | 1,165 | 0 | 100 |
| *FGF8* | 8 | 10 | 1,426 | 148 | 89.62 |
| *FGFR2* | 24 | 49 | 7,874 | 565 | 92.82 |
| *FOXE1* | 1 | 18 | 3,482 | 137 | 96.07 |
| *IRF6* | 9 | 28 | 4,694 | 648 | 86.2 |
| *MAFB* | 1 | 17 | 3,381 | 68 | 97.99 |
| *MSX1* | 2 | 11 | 1,979 | 50 | 97.47 |
| *MTHFR* | 12 | 45 | 7,402 | 884 | 88.06 |
| *MYH9* | 41 | 68 | 8,366 | 54 | 99.35 |
| *PDGFC* | 6 | 22 | 3,134 | 150 | 95.21 |
| *PVRL1* | 10 | 38 | 6,323 | 13 | 99.79 |
| *SUMO1* | 7 | 20 | 2,367 | 354 | 85.04 |
| *TGFA* | 7 | 28 | 4,575 | 156 | 96.59 |
| *TGFB3* | 7 | 21 | 3,330 | 0 | 100 |
| *VAX1* | 5 | 28 | 5,893 | 1,154 | 80.42 |
| **Total** | **216** | **501** | **80947** | **4785** | **94.09** |

**Table S3 All nonsynonymous variants found in individuals with nonsyndromic orofacial clefts**

| **Gene** | **Coding** | **Amino acid change** | **PolyPhen** | **SIFT** | **Number of cases** |
| --- | --- | --- | --- | --- | --- |
| *MTHFR* | c.1816C > T | p.R606C | Possibly damaging | Damaging | 1/103 |
| *MTHFR* | c.62G > A | p.S21N | Benign | Damaging | 1/103 |
| *MYH9* | c.5722G > A | p.D1908N | Possibly damaging | Damaging | 1/103 |
| *MYH9* | c.3676C > T | p.R1226W | Benign | Damaging | 1/103 |
| *MYH9* | c.3320G > A | p.R1107Q | Possibly damaging | Damaging | 1/103 |
| *MYH9* | c.3262G > A | p.A1088T | Possibly damaging | Damaging | 1/103 |
| *MYH9* | c.2606C > T | p.T869M | Benign | Damaging | 1/103 |
| *MYH9* | c.452A > G | p.Y151C | Possibly damaging | Damaging | 1/103 |
| *CRISPLD2* | c.119_121del | p.40_41del | — | — | 1/103 |
| *CRISPLD2* | c.1337C > G | p.A446G | Possibly damaging | Damaging | 1/103 |
| *ABCA4* | c.6498C > G | p.I2166M | Possibly damaging | Tolerated | 1/103 |
| *ABCA4* | c.4610C > T | p.T1537M | Possibly damaging | Damaging | 2/103 |
| *ABCA4* | c.4297G > A | p.V1433I | Possibly damaging | Tolerated | 1/103 |
| *ABCA4* | c.763C > T | p.R255C | Possibly damaging | Tolerated | 1/103 |
| *FOXE1* | c.1090G > A | p.G364S | Possibly damaging | Tolerated | 2/103 |
| *FGF8* | c.251C > T | p.P84L | Possibly damaging | Damaging | 1/103 |
| *FGF8* | c.250C > T | p.P84S | Possibly damaging | Damaging | 1/103 |
| *VAX1* | c.363dupT | p.C122fs | — | — | 1/103 |
| *PVRL1* | c.334T > A | p.S112T | Benign | — | 1/103 |
| *PVRL1* | c.52C > T | p.L18F | Benign | — | 2/103 |
| *FGFR2* | c.293C > T | p.T98M | Possibly damaging | Damaging | 1/103 |
| *IRF6* | c.421_423del | p.141_141del | — | — | 1/103 |
| *MYH9** | c.5188C > T | p.R1730C | Possibly damaging | Damaging | 1/103 |
| *BMP4** | c.502G > C | p.G168R | Possibly damaging | Damaging | 1/103 |
| *TGFA** | c.113C > A | p.A38E | — | — | 1/103 |
| *ABCA4** | c.5846delG | p.G1949fs | — | — | 4/103 |
| *FOXE1** | c.1058delC | p.A353fs | — | — | 2/103 |
| *PVRL1** | c.1332_1333insAGG | p.G445delinsRG | — | — | 10/103 |
| *FGFR2** | c.799A > C | p.T267P | Possibly damaging | Damaging | 2/103 |

*Nonsynonymous variants also found in normal controls.

**Table S4** All nonsynonymous variants found in normal controls

| **Gene** | **Coding** | **Amino acid change** | **PolyPhen** | **SIFT** | **Number of cases** |
| --- | --- | --- | --- | --- | --- |
| *MYH9* | c.5488C > T | p.R1830C | Possibly damaging | Damaging | 1/100 |
| *MYH9* | c.2924 A > T | p.K975M | Possibly damaging | Damaging | 1/100 |
| *CRISPLD2* | c.1036G > A | p.G346S | Possibly damaging | Damaging | 1/100 |
| *ABCA4* | c.5407G > A | p.G1803S | Possibly damaging | Damaging | 1/100 |
| *ABCA4* | c.4715C > T | p.T1572M | Possibly damaging | Damaging | 1/100 |
| *ABCA4* | c.673G > A | p.V225M | Possibly damaging | Tolerated | 1/100 |
| *ABCA4* | c.175A > C | p.K59Q | Possibly damaging | Tolerated | 1/100 |
| *VAX1* | c.833C > A | p.P278H | Possibly damaging | Tolerated | 1/100 |
| *MYH9** | c.5188C > T | p.R1730C | Possibly damaging | Damaging | 1/100 |
| *BMP4** | c.502G > C | p.G168R | Possibly damaging | Damaging | 1/100 |
| *TGFA** | c.113C > A | p.A38E | — | — | 1/100 |
| *ABCA4** | c.5846delG | p.G1949fs | — | — | 2/100 |
| *FOXE1** | c.1058delC | p.A353fs | — | — | 2/100 |
| *PVRL1** | c.1332_1333insAGG | p.G445delinsRG | — | — | 2/100 |
| *FGFR2** | c.799A > C | p.T267P | Possibly damaging | Damaging | 1/100 |

*Nonsynonymous variants also found in individuals with nonsyndromic orofacial clefts.
